# Mitochondrial Reactive Oxygen Species Enhance Alveolar Macrophage Activity against Aspergillus fumigatus but Are Dispensable for Host Protection

**DOI:** 10.1128/mSphere.00260-21

**Published:** 2021-06-02

**Authors:** Neta Shlezinger, Tobias M. Hohl

**Affiliations:** a Infectious Disease Service, Department of Medicine, Memorial Hospital, Memorial Sloan Kettering Cancer Center, New York, New York, USA; b Immunology Program, Sloan Kettering Institute, Memorial Sloan Kettering Cancer Center, New York, New York, USA; c Koret School of Veterinary Medicine, The Robert H. Smith Faculty of Agriculture, Food and Environment, The Hebrew University of Jerusalem, Rehovot, Israel; University of Georgia

**Keywords:** fungus, innate immunity, lung

## Abstract

Aspergillus fumigatus is the most common cause of mold pneumonia worldwide, and a significant cause of infectious morbidity and mortality in immunocompromised individuals. The oxidative burst, which generates reactive oxidative species (ROS), plays a pivotal role in host defense against aspergillosis and induces regulated cell death in Aspergillus conidia, the infectious propagules. Beyond the well-established role of NADP (NADPH) oxidase in ROS generation by neutrophils and other innate effector cells, mitochondria represent a major ROS production site in many cell types, though it is unclear whether mitochondrial ROS (mtROS) contribute to antifungal activity in the lung. Following A. fumigatus infection, we observed that innate effector cells, including alveolar macrophages (AMs), monocyte-derived dendritic cells (Mo-DCS), and neutrophils, generated mtROS, primarily in fungus-infected cells. To examine the functional role of mtROS, specifically the H_2_O_2_ component, in pulmonary host defense against A. fumigatus, we infected transgenic mice that expressed a mitochondrion-targeted catalase. Using a reporter of fungal viability during interactions with leukocytes, mitochondrial H_2_O_2_ (mtH_2_O_2_) was essential for optimal AM, but not for neutrophil phagocytic and conidiacidal activity in the lung. Catalase-mediated mtH_2_O_2_ neutralization did not lead to invasive aspergillosis in otherwise immunocompetent mice and did not shorten survival in mice that lack NADPH oxidase function. Collectively, these studies indicate that mtROS-associated defects in AM antifungal activity can be functionally compensated by the action of NADPH oxidase and by nonoxidative effector mechanisms during murine A. fumigatus lung infection.

**IMPORTANCE** Aspergillus fumigatus is a fungal pathogen that causes invasive disease in humans with defects in immune function. Airborne conidia, the infectious propagules, are ubiquitous and inhaled on a daily basis. In the respiratory tree, conidia are killed by the coordinated actions of phagocytes, including alveolar macrophages, neutrophils, and monocyte-derived dendritic cells. The oxidative burst represents a central killing mechanism and relies on the assembly of the NADPH oxidase complex on the phagosomal membrane. However, NADPH oxidase-deficient leukocytes have significant residual fungicidal activity *in vivo*, indicating the presence of alternative effector mechanisms. Here, we report that murine innate immune cells produce mitochondrial reactive oxygen species (mtROS) in response to fungal interactions. Neutralizing the mtROS constituent hydrogen peroxide (H_2_O_2_) via a catalase expressed in mitochondria of innate immune cells substantially diminished fungicidal properties of alveolar macrophages, but not of other innate immune cells. These data indicate that mtH_2_O_2_ represent a novel AM killing mechanism against Aspergillus conidia. mtH_2_O_2_ neutralization is compensated by other killing mechanisms in the lung, demonstrating functional redundancy at the level of host defense in the respiratory tree. These findings have important implications for the development of host-directed therapies against invasive aspergillosis in susceptible patient populations.

## INTRODUCTION

Aspergillus species are ubiquitous molds that form and disperse conidia (i.e., vegetative spores) into the air. These infectious propagules can penetrate deeply into the respiratory tract upon inhalation (1). In immunocompetent individuals, conidia are effectively cleared from the lungs by the respiratory innate immune system. Beyond the essential role of neutrophils and alveolar macrophages (AMs), monocyte-derived and plasmacytoid dendritic cells cooperate to prevent the germination of conidia into tissue-invasive hyphae (2–6). However, patients with a compromised innate immune function are at risk of developing invasive aspergillosis (IA). Aspergillus fumigatus accounts for about 65% of all invasive infections in humans, with ∼300,000 annual cases and is the most frequently encountered Aspergillus spp. in pulmonary infections (7).

The generation of the oxidative burst is a signature event associated with the activation of phagocytic cells (8) and involves the assembly and formation of a functional NADPH oxidase enzyme on the phagosomal membrane (7, 9). The requirement of reactive oxidative species (ROS) production by NADPH oxidase to protect against invasive fungal infections is underscored by the high prevalence of Aspergillus infections in patients with chronic granulomatous disease (CGD), a rare hereditary disease, in which a defect in one of the subunits of NADPH oxidase leads to a defect in ROS production by phagocytes, resulting in severe recurrent bacterial and fungal infections (10).

Previous studies from our and other groups indicate that NADPH oxidase-deficient neutrophils have significant residual conidiacidal activity *in vitro* and *in vivo* (11–13). Furthermore, most CGD patients survive years without infection, despite ubiquitous exposure to A. fumigatus conidia (14). These observations led to the discovery of NADPH oxidase-independent fungicidal mechanisms, for example, nutritional immunity which relies on the sequestration of essential transition metals by host proteins (15–18). Notably, sequestration of iron and zinc by neutrophil lactoferrin and manganese chelation by neutrophil calprotectin play essential roles in host defense against fungal pathogens (13, 19–21).

However, NADPH oxidases are not the only intracellular source of ROS. Hence, alternate ROS-dependent killing pathways may contribute to the antifungal properties of innate immune cells. Over the past decade, studies have demonstrated that mitochondrial ROS (mtROS) play multifaceted signaling functions that orchestrate innate and adaptive immunity (22–28). These include macrophage phagocytosis, the assembly of inflammasomes, cytokine production, and antigen processing (29–31). Recent studies have implicated mtROS in bacterial clearance from infected macrophages and monocyte-derived dendritic cells through the recruitment of mitochondria to pathogen-containing phagosomes (32), consistent with the idea that mtROS can augment antibacterial defenses. In contrast to bacteria, mtROS have not been integrated into an *in vivo* model of host defense against fungal pathogens.

In this study, we explored the role of mtROS in antifungal immunity against A. fumigatus. By employing a fluorescent reporter of fungal viability, we quantified the role of mtROS in conidial phagocytosis and killing by cells of respiratory innate immune system with single-encounter resolution. Using wild-type and transgenic mice that expressed a mitochondrion-localized catalase (mCAT), we demonstrated mtROS generation in a range of innate immune cells in response to Aspergillus infection and found that the H_2_O_2_ component of mtROS was crucial for optimal alveolar macrophage antifungal activity. mtH_2_O_2_ regulated conidial uptake and killing by alveolar macrophages in an NADPH oxidase-independent and cell intrinsic manner. These results implicate a specific mtROS as a regulator of AM antifungal immunity and demonstrate functional redundancy with other oxidative and nonoxidative killing systems in the lung.

## RESULTS

### Leukocytes produce mtROS in response to phagocytosed A. fumigatus conidia.

**FIG 1:**
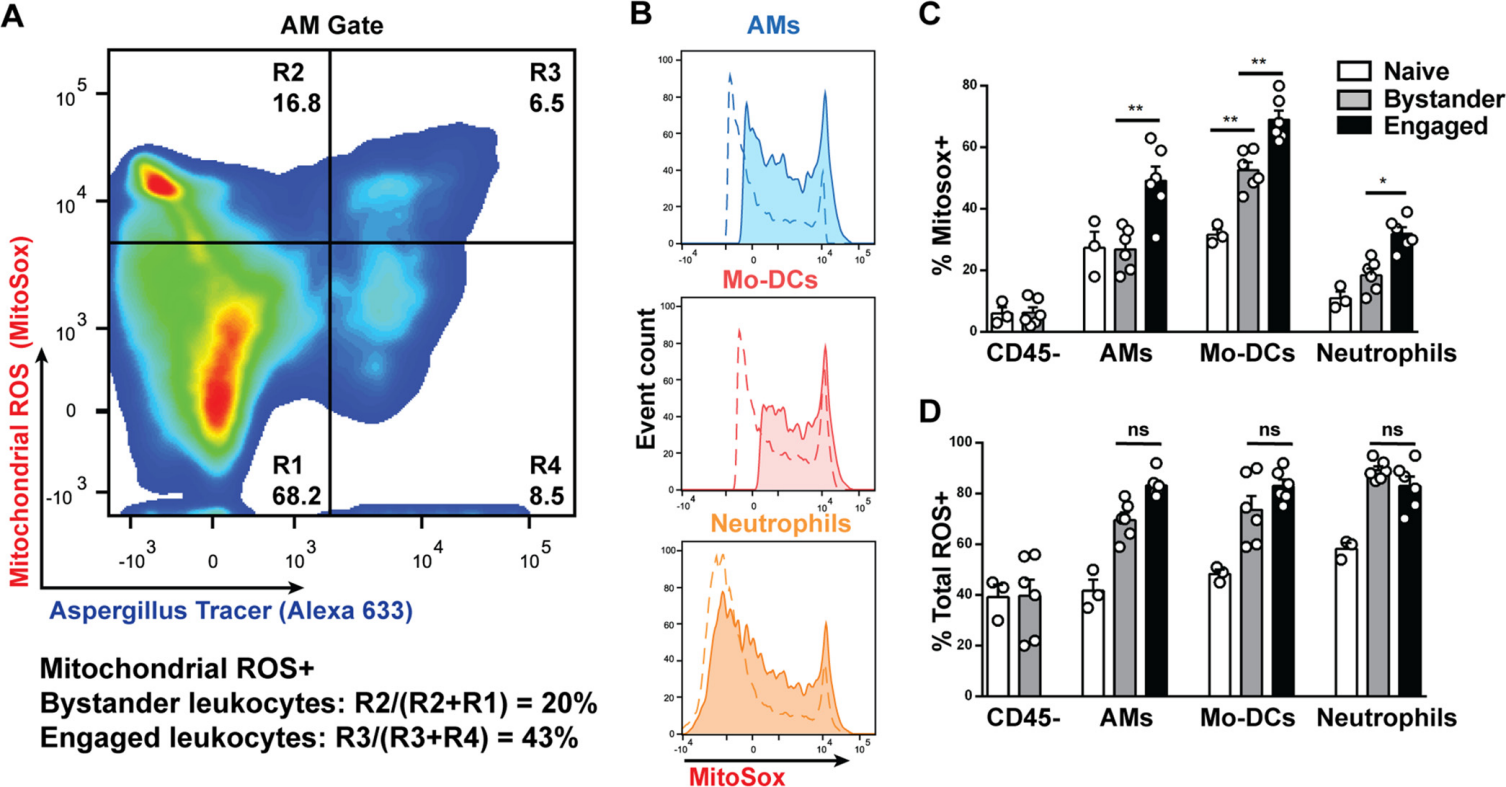
A. fumigatus conidia trigger mitochondrial ROS in lung leukocytes. C57BL/6 mice were challenged with 3 × 10^7^ A. fumigatus FLARE conidia, and single-cell lung suspensions were analyzed for mitochondrial ROS (MitoSox) and total ROS (CM-H2DCFDA) 24 h postchallenge. (A) Mitochondrial ROS staining in AF633^−^ bystander and AF633^+^ fungus-engaged alveolar macrophages (AMs). The gates indicate the frequencies of mtROS-negative (R1 and R4) and mtROS-positive (R2 and R3) bystander (R1 and R2) or fungus-engaged (R3 and R4) AMs. (B) Histogram of mitochondrial ROS in bystander (broken line) or fungus-engaged (continuous line) alveolar macrophages, Mo-DCs, and lung neutrophils. (C and D) Mitochondrial ROS (C) and total ROS (D) in naive (white bars), bystander (gray bars), and engaged (black bars) CD45^−^ nonhematopoietic, alveolar macrophages, Mo-DCs, and neutrophils. Data shown represent means ± SEM (error bars) from two independent experiments (*n* = 6 for infected mice, *n* = 3 for naive mice). Statistical analysis was performed with a one-way ANOVA (*P* < 0.001) followed by a *post hoc* Tukey HSD analysis. Statistical significance: *, *P* < 0.05; **, *P* < 0.01; ns, not significant.

To determine whether Aspergillus interactions stimulate mtROS production in lung leukocytes, C57BL/6 mice were challenged with A. fumigatus conidia, and mtROS was quantified in single-cell lung suspensions (Fig. 1A). Fungus-engaged and bystander lung leukocytes were distinguished by the use of fluorescent Aspergillus reporter (FLARE) conidia that were covalently labeled with the fluorescent tracer Alexa Fluor 633 (AF633) (12). Conidial uptake by alveolar macrophages (AMs) triggered mtROS induction, as judged by MitoSox staining of fungus-engaged cells (AF633-positive [AF633^+^]) compared with bystander AMs and with AMs isolated from naive mice (Fig. 1B to D). Similarly, conidial uptake triggered mtROS induction in lung monocyte-derived dendritic cells (Mo-DCs) and neutrophils (Fig. 1B to D). In contrast, mtROS induction was not observed in CD45^−^ nonhematopoietic cells isolated from A. fumigatus-infected lungs. These data demonstrate that conidium-leukocyte interactions induce, at least in part, mtROS induction during respiratory A. fumigatus challenge.

### Mitochondrial ROS mediate AM phagocytic and fungicidal activity in the lung.

To examine the functional role of mtROS in antifungal immunity, we utilized well-characterized transgenic mice that expressed a mitochondrion-localized human catalase (mCAT^Tg/+^) and thus neutralized mtH_2_O_2_ (33). mCAT^Tg/+^ mice exhibit significantly lower mitochondrial H_2_O_2_ levels, reduced age-related oxidative damage, and an extended life span compared to nontransgenic littermate controls (33). In the first series of experiments, we measured the leukocyte intrinsic role of mtH_2_O_2_ in conidial uptake and conidial killing by generating mixed bone marrow chimeric mice, in which irradiated CD45.1^+^ CD45.2^+^ recipients were reconstituted with a 1:1 ratio of CD45.2^+^ mCAT^Tg/+^ and CD45.1^+^ wild-type congenic bone marrow cells (see Fig. S1 in the supplemental material). The properties of CD45.2^+^ mCAT^Tg/+^ and CD45.1^+^ wild-type leukocytes were thus compared in the same host inflammatory environment (Fig. S2).

10.1128/mSphere.00260-21.1 FIG S1 Generation of mixed bone marrow chimeric mice. Schematic illustration of the strategy used for generation, challenge, and analysis of mixed BM chimeric mice. Recipient CD45.1^+^ CD45.2^+^ mice were irradiated and reconstituted with equal numbers of donor BM cells of the indicated genotypes (white and gray circles or blue and purple circles) to generate (1) mixed BM chimeric mice that contain WT and mitochondrial catalase (mCAT)-expressing leukocytes or (2) mixed BM chimeric mice that contain p91^phox−/−^ leukocytes that either express mitochondrial catalase (mCAT^Tg/+^) or not. Download FIG S1, PDF file, 0.2 MB. Copyright © 2021 Shlezinger and Hohl. 2021 Shlezinger and Hohl. https://creativecommons.org/licenses/by/4.0/ This content is distributed under the terms of the Creative Commons Attribution 4.0 International license.

10.1128/mSphere.00260-21.2 FIG S2 Reconstitution of leukocytes in mixed bone marrow chimeric mice. CD45 chimerism in the blood and bone marrow in recipient mice 8 weeks after reconstitution with CD45.1^+^ wild type and CD45.2^+^ mCAT^Tg/+^ donor BM cells or with CD45.1^+^ p91phox^−/−^ and CD45.2^+^ p91phox^−/−^ mCAT^Tg/+^ BM donor cells. The proportions of CD45.1^+^ and CD45.2^+^ cells are indicated in recipient mice, as determined by flow cytometric analysis. *n* ≥ 9 per group pooled from two independent experiments. Download FIG S2, PDF file, 0.1 MB. Copyright © 2021 Shlezinger and Hohl. 2021 Shlezinger and Hohl. https://creativecommons.org/licenses/by/4.0/ This content is distributed under the terms of the Creative Commons Attribution 4.0 International license.

**FIG 2:**
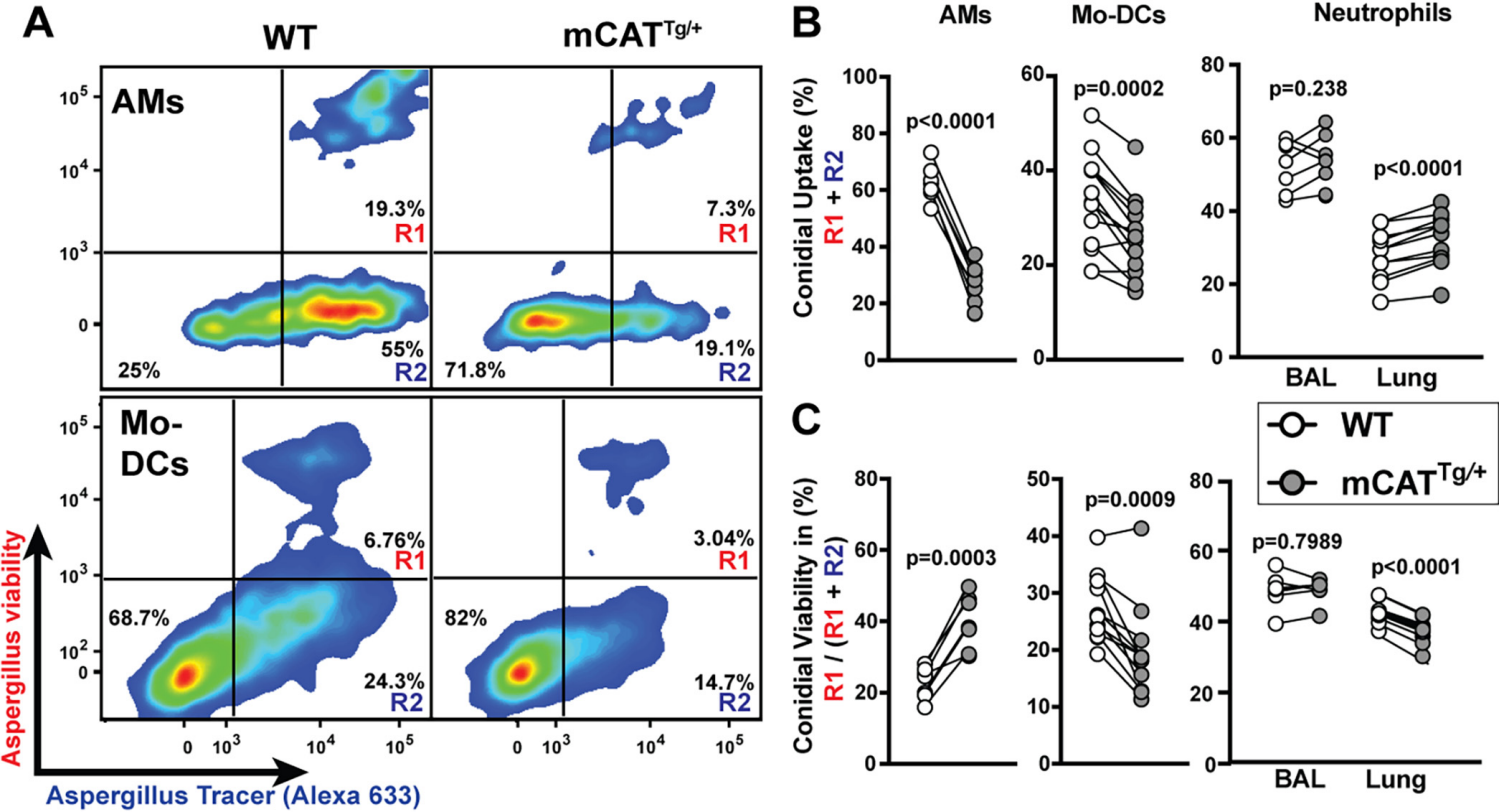
Impact of mtH_2_O_2_ neutralization on AMs, Mo-DCs, and neutrophil conidial uptake and killing in the lung. Mixed BM chimeric mice were infected with 3 × 10^7^ FLARE conidia intranasally. (A) Representative plots showing WT or mCAT-expressing AMs and lung Mo-DCs harvested from mixed bone marrow chimeric mice 24 h postinfection (p.i.), analyzed on the basis of dsRed (Aspergillus viability) and AF633 (Aspergillus tracer) fluorescence. R1, leukocytes with live conidia; R2, leukocytes with dead conidia. (B and C) The scatterplots indicate conidial uptake (R1 + R2) (B) by and intraphagosomal conidial viability [R1/(R1 + R2)] (C) in indicated leukocyte subsets. The lines indicate paired data sets isolated from a single mixed chimeric mouse. *n* ≥ 9 per group pooled from yeoindependent experiments. A paired *t* test was used for the statistical analysis.

Mixed chimeric mice were infected with FLARE conidia that, in addition to the AF633 tracer fluorophore, also encoded a dsRed transgene which acted as a marker of fungal viability (12). The dsRed tracer fluorophore distinguished AF633^+^ fungus-engaged leukocytes that contained either live (dsRed^+^) or killed (dsRed^−^) conidia, as shown for AMs in Fig. 2A. The frequency of fungus-engaged AF633^+^ AMs (Fig. 2A, conidial uptake = R1 + R2) was vastly reduced in mCAT-expressing cells (mCAT^Tg/+^, 35% ± 3%) compared to WT cells (70% ± 2%; Fig. 2B) at 24 h postinfection. Similar results were observed in mCAT-expressing Mo-DCs compared to wild-type (WT) Mo-DCs (Fig. 2A, lower panel, and Fig. 2B), consistent with a conidial uptake defect in mCAT^Tg/+^ AMs and Mo-DCs compared to wild-type counterparts isolated from the same inflammatory milieu. Moreover, the frequency of fungus-engaged AMs that contained live conidia was increased in mCAT^Tg/+^ AMs (38% ± 3%) compared to WT (22% ± 2%) AMs [Fig. 2A, intraleukocyte conidial viability = R1/(R1 + R2)], indicating an AM intrinsic defect in intracellular conidial killing when mtH_2_O_2_ were neutralized (Fig. 2C). Notably, mitochondrial catalase expression did not interfere with lung and bronchoalveolar lavage fluid (BALF) neutrophil conidiacidal activity (Fig. 2B and C). In fact, mCAT^Tg/+^ neutrophils exhibited a modest increase in conidial uptake and conidial killing. Surprisingly, mCAT^Tg/+^ Mo-DCs also exhibited improved conidial killing. Collectively, these findings indicate that mtH_2_O_2_ differentially regulated AM, Mo-DC, and neutrophil phagocytic and fungicidal activity during A. fumigatus infection, with an essential role for optimal AM antifungal activity.

**FIG 3:**
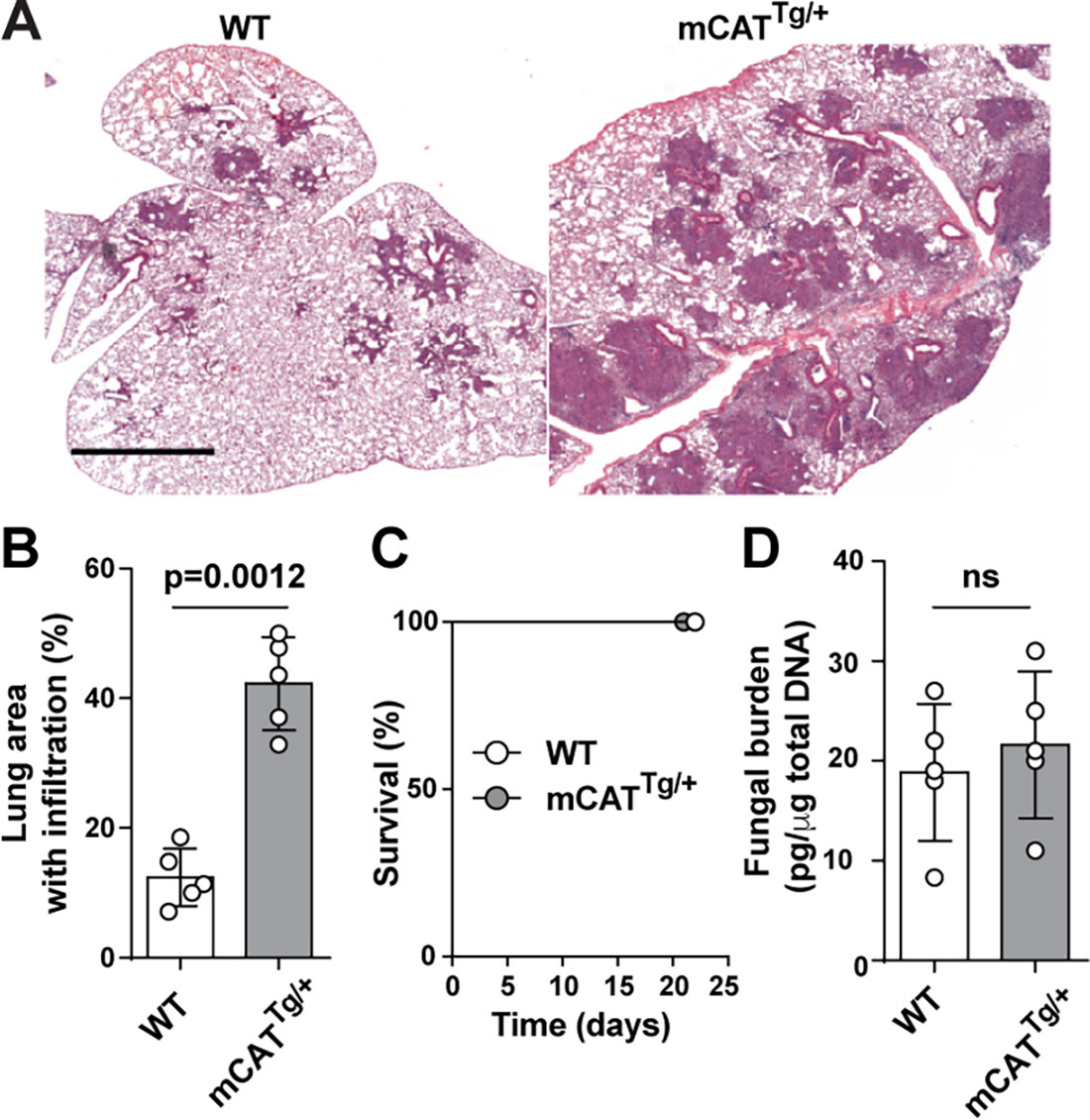
mtH_2_O_2_ neutralization regulates lung inflammation but is dispensable for A. fumigatus susceptibility. Immunocompetent C57BL/6 and mCAT^Tg/+^ mice were challenged with 8 × 10^7^ Af293 conidia. (A) Representative micrographs of hematoxylin and eosin-stained lung sections from two independent experiments (bar, 2 mm). (B) Quantitative morphometric analysis of lung consolidation at day +3 postinfection. (C) Kaplan-Meier survival analysis. (D) Lung fungal burden (fungal DNA) at day +3 postinfection, according to murine genotype. Data shown represent means ± SEM (error bars) (*n* = 5 for panel B, *n* = 10 for panel C, *n* = 6 for panel D). For statistical analysis, *t* test was used for the data in panels B and D, and log rank (Mantel-Cox) test was used for the data in panel C.

To define the role of mtH_2_O_2_ on infectious outcomes following A. fumigatus infection, we compared the histopathology and mortality in mCAT^Tg/+^ mice and nontransgenic littermate controls. Lung histopathology of mCAT^Tg/+^ mice 3 days postinfection revealed multifocal areas of necrosis and inflammation that affected >40% of the parenchyma, while control mice had moderate multifocal inflammation that involved ∼10% of the parenchyma (Fig. 3A and B). Despite widespread inflammatory lesions in mCAT^Tg/+^ lung sections, evidence of conidial germination and hyphal tissue invasion was not observed (Fig. S3).

10.1128/mSphere.00260-21.3 FIG S3 Representative images of GMS-stained murine lung sections following Aspergillus challenge. Immunocompetent C57BL/6 wild-type and mCAT^Tg/+^ mice were challenged with 8 × 10^7^ Af293 conidia, and p91phox^−/−^ and p91phox^−/−^ mCAT^Tg/+^ mice were challenged with 5 × 10^4^ Af293 conidia. Lungs were harvested 72 h p.i. and stained with Grocott-Gomori’s methenamine-silver (GMS). Representative micrographs of lung sections stained with GMS (×40). Download FIG S3, PDF file, 0.3 MB. Copyright © 2021 Shlezinger and Hohl. 2021 Shlezinger and Hohl. https://creativecommons.org/licenses/by/4.0/ This content is distributed under the terms of the Creative Commons Attribution 4.0 International license.

To determine whether catalase-dependent mtH_2_O_2_ neutralization influenced murine susceptibility to A. fumigatus, immunocompetent mCAT^Tg/+^ and littermate control mice were challenged with 8 × 10^7^ A. fumigatus Af293 conidia and monitored for survival (Fig. 3C). Mice from both genotypes all survived the challenge, and consistent with this finding, the lung fungal burden at 3 days postinfection, as measured by fungal DNA content, was not affected by mitochondrion-targeted H_2_O_2_ neutralization (Fig. 3D). These findings indicate that mtH_2_O_2_ is not essential for protection against respiratory A. fumigatus challenge with the low virulence Af293 strain.

**FIG 4:**
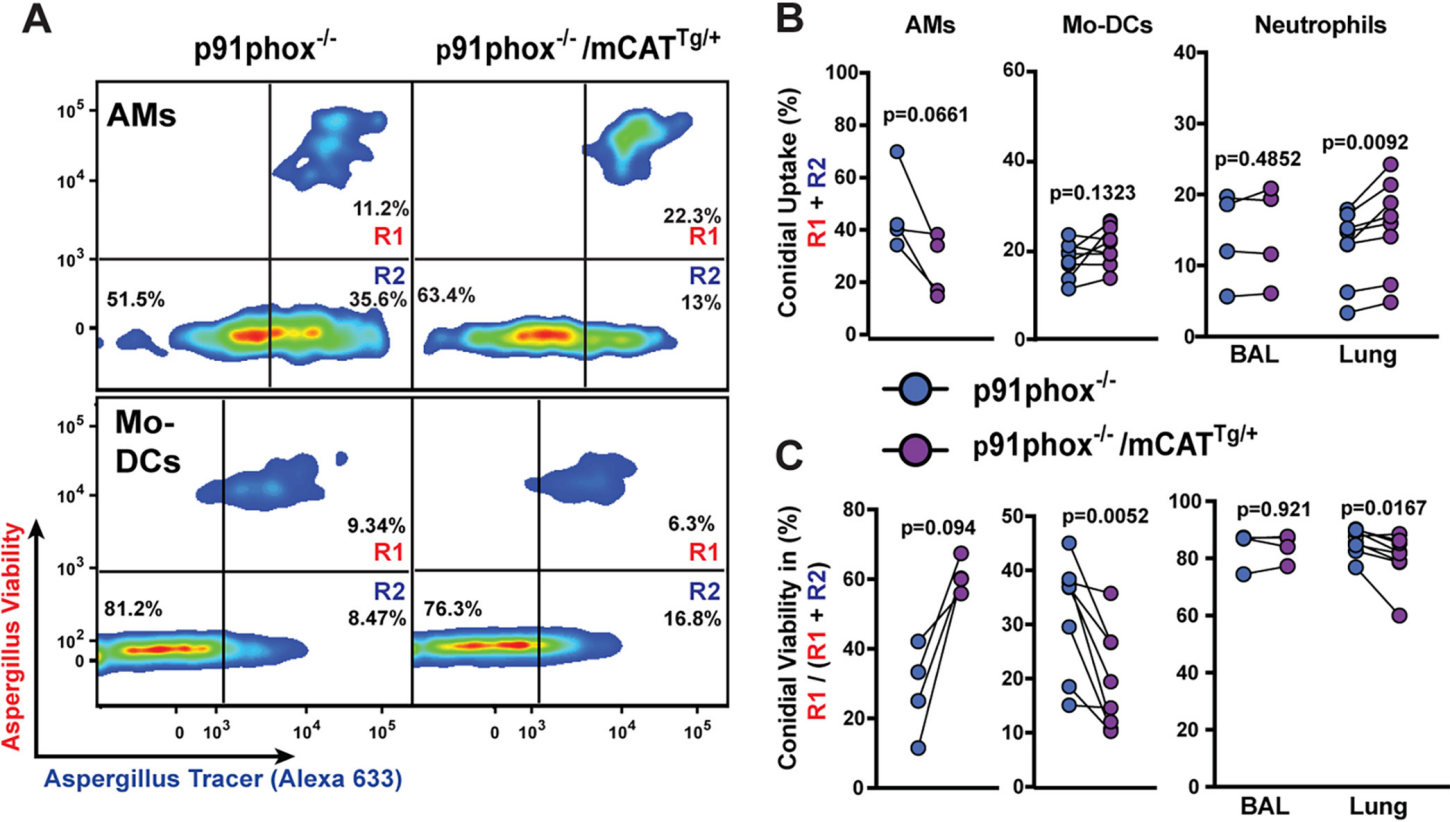
Impact of mtH_2_O_2_ neutralization on leukocyte conidial uptake and killing in NADPH oxidase-deficient mice. Mixed BM chimeric mice were infected with 3 × 10^7^ FLARE conidia intranasally. (A) Representative plots showing *p91phox*^−/−^ or mCAT-expressing *p91phox*^−/−^ (mCAT^Tg/+^ p91phox^−/−^) BAL fluid alveolar macrophages and lung Mo-DCs harvested from mixed bone marrow chimeric mice 24 h p.i., analyzed on the basis of dsRed (viability) and AF633 (tracer) fluorescence. R1, leukocytes with live conidia; R2, leukocytes with dead conidia. (B and C) The scatterplots indicate conidial uptake (R1 + R2) (B) by and intraphagosomal conidial viability [R1/(R1 + R2)] (C) in indicated leukocyte subsets. The lines indicate paired data sets isolated from a single mixed chimeric mouse. *n* ≥ 6 per group pooled from two independent experiments. A paired *t* test was used for statistical analysis.

One possible explanation for these results is that products of NADPH oxidase can compensate for the mitochondrion-targeted catalase-catalyzed H_2_O_2_ breakdown. To test this hypothesis, we crossed the mCAT transgene to the *p91phox*^−/−^ background and generated mixed bone marrow chimeric mice to compare the cell intrinsic fungicidal activities of CD45.1^+^ *p91phox*^−/−^ and CD45.2^+^ *p91phox*^−/−^ mCAT^Tg/+^ leukocytes in the same lung (Fig. S1 and S2), as outlined above. Following infection with FLARE conidia, AM conidial uptake was reduced in *p91phox*^−/−^ mCAT^Tg/+^ AMs (34% ± 5%) compared to *p91phox*^−/−^ (50% ± 6%) counterparts at 24 h postinfection (Fig. 4A and B). In addition, intraleukocyte conidial viability was higher in *p91phox*^−/−^ mCAT^Tg/+^ AMs (60% ± 2%) compared to p*91phox*^−/−^ (28% ± 7%) counterparts that had intact mtH_2_O_2_ levels, though the difference did not reach statistical significance (Fig. 4C). The previously observed mCAT-dependent defect in conidial uptake by Mo-DCs was not observed in the context of NADPH oxidase deficiency (Fig. 4B). Mo-DC and neutrophil intracellular conidial killing in *p91phox*^−/−^ mice was slightly higher with mCAT transgene expression compared to nontransgenic counterparts, consistent with the idea that mtH_2_O_2_ did not contribute to conidiacidal activity in these cell types (Fig. 4C).

**FIG 5:**
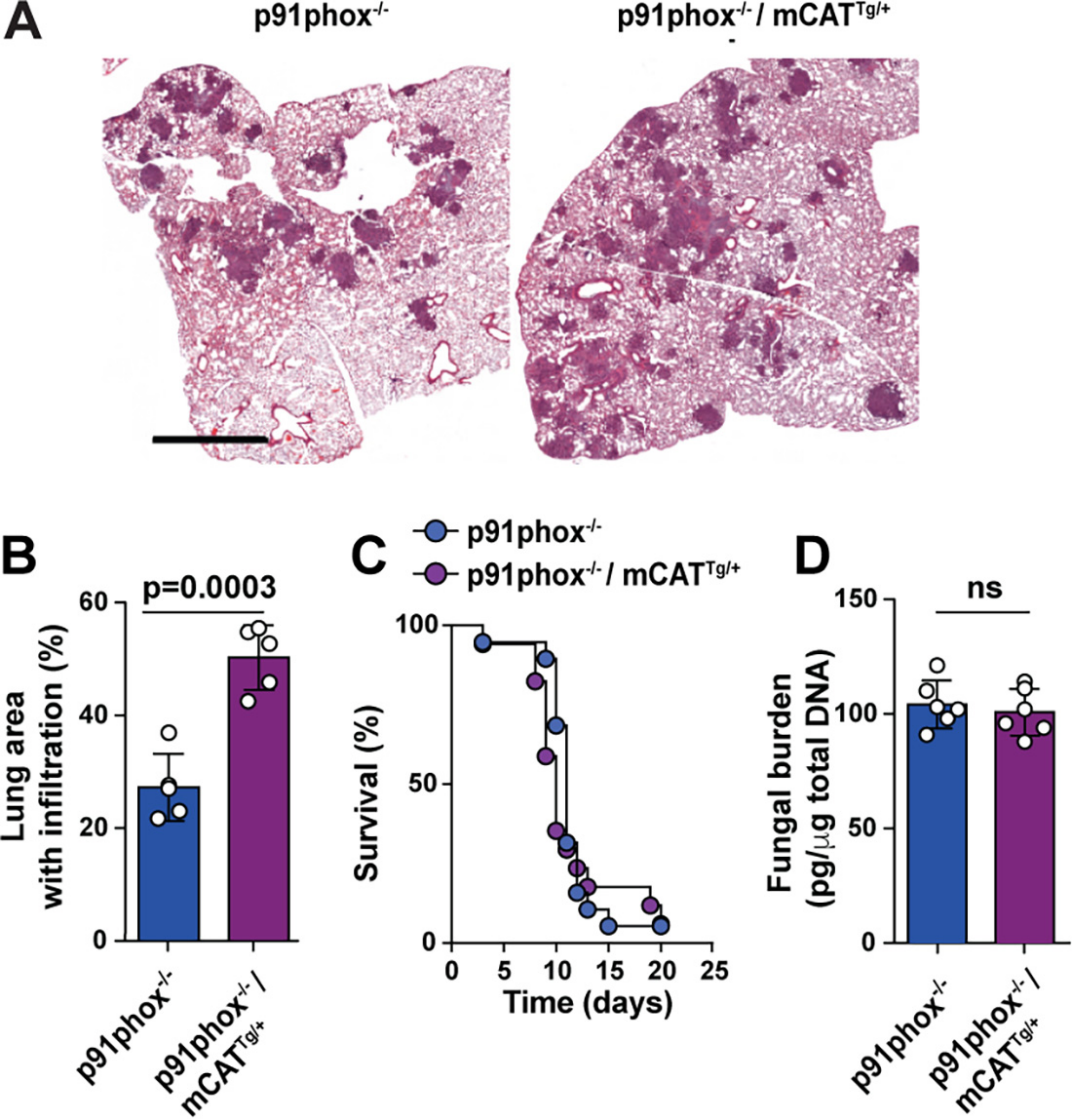
Impact of mtH_2_O_2_ neutralization on lung inflammation, survival, and fungal growth in NADPH oxidase-deficient mice. *p91phox*^−/−^ and mCAT^Tg/+^ p91phox^−/−^ mice were challenged with 5 × 10^4^ Af293 conidia. (A) Micrographs of lung sections stained with hematoxylin and eosin from two independent experiments (bar, 2 mm). (B) Quantitative morphometric analysis of lung consolidation at day +3 postinfection. (C) Kaplan-Meier survival analysis. (D) Lung fungal burden (fungal DNA) day +3 postinfection, according to murine genotype. Data shown represent means ± SEM (*n* = 5 for panel B, *n* = 19 for p91phox^−/−^ and *n* = 17 mCAT^Tg/+^ p91phox^−/−^ in panel C, and *n* = 6 for panel D). For statistical analysis, *t* test was used for the data in panels B and D, and log rank (Mantel-Cox) test was used for the data in panel C.

To determine whether mtH_2_O_2_ was essential for host defense in the context of NADPH oxidase deficiency, *p91^phox−/−^* mCAT^Tg/+^ and nontransgenic *p91^phox−/−^* control mice were challenged with 5 × 10^4^ conidia and monitored for survival, lung pathology, and fungal burden (Fig. 5). In line with previous studies (10), *p91phox*^−/−^ mice succumbed to infection (Fig. 5A and B). Although mtH_2_O_2_ neutralization in the *p91phox*^−/−^ background further exacerbated lung inflammation, this finding was not associated with increased mortality and a higher lung fungal burden compared to *p91phox*^−/−^ mice (Fig. 5C and D).

## DISCUSSION

In this study, we demonstrated that A. fumigatus infection induced mtROS production in a range of innate immune effector cells during pulmonary infection. In AMs, the mtROS constituent H_2_O_2_ contributed to fungicidal activity against conidia, since catalase-mediated neutralization of mtH_2_O_2_ diminished their antifungal activity *in vivo*. These findings mirror and extend a study of cultured macrophages, recently published by Hatinguais and colleagues (34). In this work, pharmacologic inhibition of the macrophage mitochondrial reverse electron transport chain reduced Aspergillus metabolic activity and regulated Aspergillus-triggered cytokine production *in vitro* (34).

Our findings clarify and expand previous studies that highlight context-specific roles for AM function in host defense. In a prior study, clodronate liposome-mediated AM depletion did not facilitate the development of lethal invasive aspergillosis (3), while another study reported an increase in lung fungal burden in clodronate liposome-treated mice (35). Previous work suggested that AM fungicidal activity was similar in NADPH oxidase-sufficient and -deficient cells, raising questions about relevant alternative conidial killing mechanisms (12, 36). In support of this notion, many bacterial, protozoan, and fungal pathogens, including Candida albicans, Cryptococcus neoformans, and A. fumigatus, subvert the phagosomal oxidative burst by interfering with NADPH oxidase complex assembly or by utilizing detoxification mechanisms, yet phagocytes retain significant microbicidal activity *in vivo*, indicating the presence of additional antifungal effectors (37–42). This study provides evidence for mtH_2_O_2_ as a bona fide AM effector molecule against A. fumigatus conidia in the lung. The precise contribution and interdependence of NADPH oxidase and mtROS to AM anti-Aspergillus activity remain to be determined, though it is notable that both rotenone- or carbonyl cyanide 4-(trifluoromethoxy) phenylhydrazone (FCCP)-treated macrophages *in vitro* (34) and mtH_2_O_2_-neutralized AMs *in vivo* both exhibit a clear trend to decreased antifungal activity when NADPH oxidase is either pharmacologically blocked (i.e., diphenyleneiodonium chloride [DPI]) or genetically ablated.

Previous studies showed that mitochondria regulate Salmonella and lipopolysaccharide (LPS)-induced Toll-like receptor (TLR) signaling and bacterial killing in murine bone marrow-derived macrophages (BMDMs) (32). TLR activation resulted in the recruitment of mitochondria to macrophage phagosomes and in enhancement of mtROS production. A subsequent study corroborated these findings in macrophage-mediated defense against methicillin-resistant Staphylococcus aureus (MRSA) and further revealed that, upon infection, endoplasmic reticulum (ER) stress stimulated mtROS production and delivery to bacterium-containing phagosomes via mitochondrion-derived vesicles (43). In agreement with our observations, expression of mitochondrial catalase or superoxide dismutase-2 (Sod2) depletion led to reduced macrophage bactericidal activity. However, it remained unknown whether mtROS constituents, individually or collectively, were essential for survival against systemic or compartmentalized infections has not been studied in animal models.

Using mCAT^Tg/+^ mice, we found that mtH_2_O_2_ neutralization did not reduce host survival or lung fungal burden in otherwise immunocompetent mice or in *p91phox^−/−^* mice. These data supported the idea that mtH_2_O_2_ did not contribute substantially to fungal killing in innate immune cells beyond AMs, even in the absence of NADPH oxidase activity. We cannot exclude the possibility that mitochondrion-localized catalase may exhibit different degrees of mtH_2_O_2_ neutralization and downstream impact on effector functions in AMs compared to other immune cell populations.

While the requirement of AMs for protection against aspergillosis can be functionally compensated in murine models of disease, neutrophils are essential for host defense and have the capacity to inhibit germination and kill conidia and hyphae *in vivo* (3, 5, 12, 44, 45). In patients with functional or numeric neutrophil defects, the role of AMs in host defense against A. fumigatus is likely accentuated, given the loss of cellular redundancy (6). Thus, we were surprised that functional impairment of mCAT^Tg/+^ AMs in *p91^phox−/−^* mice did not result in a higher lung fungal burden or in more rapid disease development. One possibility for this finding is that AM mtH_2_O_2_ is particularly relevant for host defense against conidia but less effective against hyphae and products of germination which are prevalent in *p91^phox−/−^* mice (46). In this model, once hyphae are formed, fungal killing is driven primarily by neutrophils that were not affected by mtH_2_O_2_ neutralization (3, 47). A study of zebrafish larvae demonstrated that neutrophil killing required conidial germination, while macrophages inhibited conidial germination and attenuated neutrophil recruitment and neutrophil-mediated killing (47). This model may align with our data showing that mtH_2_O_2_ neutralization rendered AMs dysfunctional with regard to fungicidal activity, resulting in increased conidial uptake and killing by lung neutrophils.

The current study was conducted using the widely used Af293 strain, a clinical isolate with low virulence characteristics. It is possible that the role of mtH_2_O_2_ in host defense may be greater, equal, or diminished with other A. fumigatus strains (e.g., CEA10) that have the capacity to germinate more rapidly and act in a more virulent manner than Af293 yet trigger more pronounced neutrophilic inflammation in the murine lung as well (48). Collectively, experiments in this study and in reference 34 support the idea that mtROS components enhance alveolar macrophage antifungal activity and that this process can act against different Aspergillus strains (Af293 and CEA10). Future work should focus on integrating mtROS in pulmonary host defense in a different model of disease, e.g., in neutropenic or in corticosteroid-treated mice, and in the context of a range of clinical A. fumigatus isolates. In conclusion, our studies reveal that mtH_2_O_2_ is dispensable for protection against A. fumigatus infection in an immunocompetent and CGD model of A. fumigatus infection. However, mtROS do have a functional role in regulating and boosting AM antifungal activity against inhaled conidia. Further studies will be needed to address the *in vivo* role and full range of mtROS against a broad range of inhaled fungal and nonfungal pathogens.

## MATERIALS AND METHODS

### Mice, animal care, and ethics statement.

C57BL/6 mice (Jackson Laboratories, strain 000664; CD45.2^+^), C57BL/6.SJL mice (Charles River Laboratories, strain 564; CD45.1^+^), C57BL/6.mCAT mice (mCAT^Tg/+^; Jackson Laboratories, strain 016197; CD45.2^+^), and *p91phox*^−/−^ mice (Jackson Laboratories, strain 002365) were bred in the Memorial Sloan Kettering Cancer Center (MSKCC) Animal Vivarium. mCAT^Tg/+^ mice were crossed to *p91phox*^−/−^ mice to generate *p91phox^−/−^* mCAT^Tg/+^ mice. Lethally irradiated (9.5 Gy) F1 progeny (from cross of C57BL/6 and C57BL/6.SJL strains) were reconstituted with 1 × 10^6^ to 2.5 × 10^6^ C57BL/6.SJL and C57BL/6.mCAT or *p91phox*^−/−^ and *p91phox*^−/−^.mCAT BM cells, treated with enrofloxacin in the drinking water for 21 days to prevent bacterial infections, and rested for 6 to 8 weeks prior to use. All animal experiments were conducted with sex- and age-matched mice and performed with approval from MSKCC Institutional Animal Care and Use Committee (protocol number 13-07-008). Animal studies were compliant with all applicable provisions established by the Animal Welfare Act and the Public Health Services Policy on the Humane Care and Use of Laboratory Animals.

### Aspergillus fumigatus culture and infection model.

A. fumigatus Af293 and Af293-dsRed (12) strains were cultured on glucose minimal medium slants at 37°C for 4 to 7 days prior to harvesting conidia for experimental use. To generate FLARE conidia, briefly, 7 × 10^8^ Af293-dsRed conidia were rotated in 10 μg/ml Biotin XX, SSE in 1 ml of 50 mM carbonate buffer (pH 8.3) for 2 h at 4°C, incubated with 20 μg/ml Alexa Fluor 633 succinimidyl ester at 37°C for 1 h, resuspended in phosphate-buffered saline (PBS) and 0.025% Tween 20 for use within 24 h (12). To infect mice with 30 to 60 million A. fumigatus cells, conidia were resuspended in PBS and 0.025% Tween 20 at a concentration of 0.6 × 10^9^ to 1.2 × 10^9^/ml cells, and 50 μl of cell suspension was administered via the intranasal route to mice anesthetized by isoflurane inhalation.

### Reagents and antibodies.

Chemical and cell culture reagents were purchased from Thermo Scientific and Gibco, unless noted otherwise. Voriconazole (Pfizer) was obtained from the Memorial Sloan Kettering Cancer Center pharmacy and dissolved in sterile PBS at 10 mg/ml and stored at −80°C. Fluorescent antibodies were from Tonbo or eBioscience.

### Fungal growth and culture.

All A. fumigatus strains were grown on glucose minimal media that contains 1% glucose, salt solution, and trace minerals (22). Conidia were harvested by flooding 5- to 10-day-old plates or slants with 10 ml PBS and 0.025% Tween 20, briefly vortexing sealed plates or slants, and by filtering the conidial suspensions twice through a 40-μm nylon cell strainer. Resting conidia were labeled with Alexa Fluor 633 as previously described (10).

### qPCR.

Lung fungal burdens in animals were determined by quantitative real-time PCR (qPCR) using methods reported by Bowman et al. (49). Briefly, lungs were homogenized using 2.5-mm sterile beads in sterile saline with a bead beater homogenizer (Mini Bead beater; BioSpec, Bartlesville, OK). DNA was then extracted from 90 μl of lung homogenate with the DNeasy tissue kit (Qiagen, Valencia, CA) according to the manufacturer’s instructions. Recovered DNA in 200 μl of elution buffer was then stored at −80°C until analysis. DNA samples were analyzed in duplicate using an ABI PRISM 7000 sequence detection system (Applied Biosystems, Inc., Foster City, CA) with primers and dually labeled fluorescent hybridization probes specific for the A. fumigatus 18S rRNA gene (GenBank accession no. AB008401). The cycle threshold (*C_T_*) of each sample, which identifies the cycle number during PCR when fluorescence exceeds a threshold value determined by the software, was then interpolated from a six-point standard curve of *C_T_* values prepared by spiking naive uninfected mouse lungs with 1 × 10^2^ to 1 × 10^7^ A. fumigatus Af293 conidia. Results were reported as A. fumigatus DNA conidial equivalents. Lungs from sham-infected mice were used as a specificity control in each experiment.

### Analysis of infected mice.

Mice were infected via the intratracheal route as described previously (2). Bronchoalveolar lavage (BAL) fluid and lung suspensions were prepared for flow cytometry as described previously (11). Briefly, lung digest and, if applicable, BAL fluid cells were enumerated and stained with fluorophore-conjugated antibodies for flow cytometric analysis on a BD LSR II. Neutrophils were identified as CD45^+^ CD11b^+^ Ly6C^lo^ Ly6G^+^ cells, alveolar macrophages as CD45^+^ CD11b^+^ CD11c^+^ Ly6G- siglecF^+^ cells, and Mo-DCs as CD45^+^ CD11b^+^ CD11c^+^ Ly6G- Ly6C^hi^ major histocompatibility complex (MHC) class II^+^ cells.

### Histology.

After euthanasia, lungs were inflated with 1 ml of 10% neutral buffered formalin, removed en bloc after tracheal ligation, preserved in 10% neutral buffered formalin for 24 h at 4˚C, and subsequently embedded in paraffin. Lung tissue sections (5 mm) were stained with hematoxylin and eosin (H&E) and Gomori’s methenamine-silver (GMS).

### ROS measurements.

Total intracellular H_2_O_2_ levels and mitochondrial superoxide levels were measured as previously described (2, 32). Briefly, lung cell single-cell suspensions were washed with PBS and then incubated with MitoSox (to measure mitochondrial superoxide) and/or CM-H2DCFDA [5- (and 6)-chloromethyl-2',7'-dichlorodihydrofluorescein diacetate, acetyl ester] (to measure total cellular H_2_O_2_) (Invitrogen) at 5 μM final concentration in HBSS buffer for 15 to 30 min at 37˚C. Cells were washed with warmed PBS three times, removed from plates with cold PBS containing 0.5 mM EDTA by pipetting, pelleted at 1,500 rpm for 5 min, immediately resuspended in cold PBS containing 1% fetal bovine serum (FBS), and subjected to fluorescence-activated cell sorting (FACS) analysis. Unstained controls were treated similarly, except that treatments and dyes were omitted. Flow cytometry data were analyzed using FlowJo software.

### Statistical analysis.

All statistical analyses were performed using GraphPad Prism version 6.01 (GraphPad Software Inc.) and display experimental data of at least two independent experiments performed in triplicate, displaying the means and standard errors of means (SEM) throughout the manuscript, except for animal-related experiments, which display means ± 95% confidence intervals. The following statistical tests were applied: comparisons between three or more groups, one-way analysis of variance (ANOVA); comparison between two groups, Student’s *t* test; animal survival curves, log rank test. Statistical significance was accepted at *P* < 0.05.
