# Supplementary material for: Mitochondrial Reactive Oxygen Species Enhance Alveolar Macrophage Activity against Aspergillus fumigatus but Are Dispensable for Host Protection
Source: mSphere. 2021 Jun 2;6(3):e00260-21. doi: 10.1128/mSphere.00260-21 (PMC8265640; doi:10.1128/mSphere.00260-21)
Supplement: FIG S1 [file msphere.00260-21-sf001.pdf]

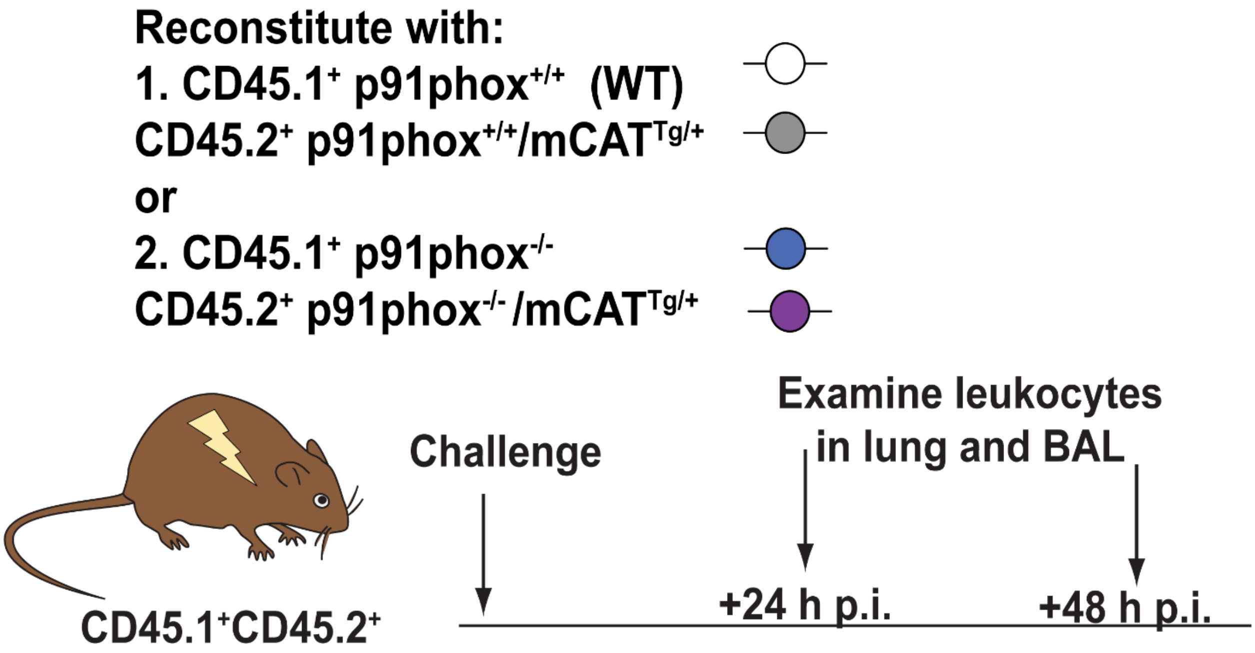
Reconstitute with:
1. CD45.1^{+} p91phox$^{+/+}$ (WT)
CD45.2^{+} p91phox$^{+/+}$/mCAT$^{Tg/+}$
or
2. CD45.1^{+} p91phox$^{-/-}$
CD45.2^{+} p91phox$^{-/-}$ /mCAT$^{Tg/+}$
CD45.1^{+}CD45.2^{+}
Challenge
Examine leukocytes in lung and BAL
+24 h p.i.
+48 h p.i.

Figure S1
