# Supplementary figures and images for: Mitochondrial Reactive Oxygen Species Enhance Alveolar Macrophage Activity against Aspergillus fumigatus but Are Dispensable for Host Protection
Source: mSphere. 2021 Jun 2;6(3):e00260-21. doi: 10.1128/mSphere.00260-21 (PMC8265640; doi:10.1128/mSphere.00260-21)

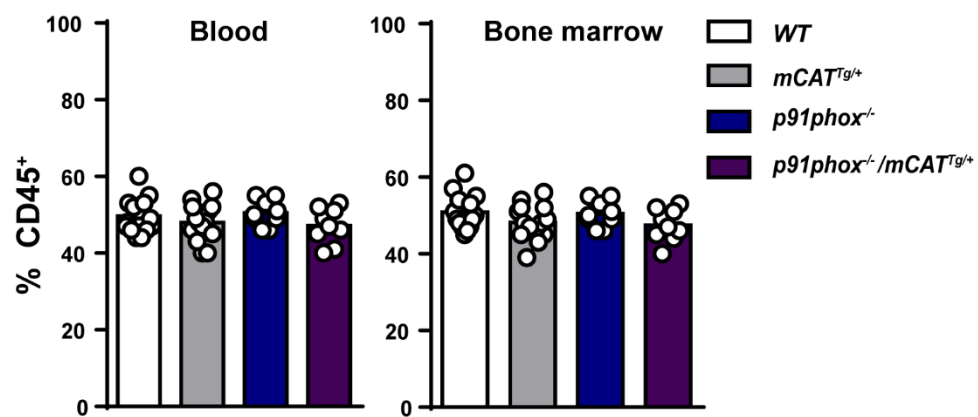

Figure S2

Supplement: FIG S2 [file msphere.00260-21-sf002.pdf]

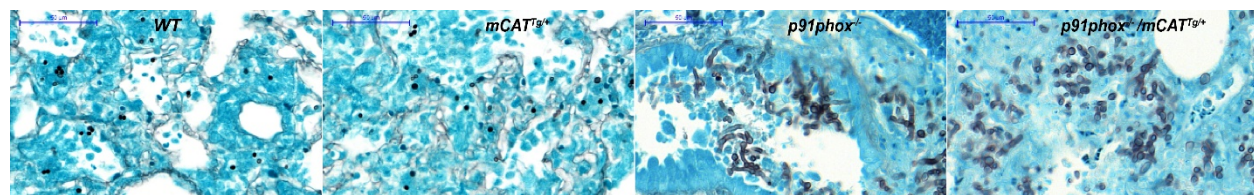

**Figure S3**

Supplement: FIG S3 [file msphere.00260-21-sf003.pdf]
